# Effect of discriminative plant-sugar feeding on the survival and fecundity of *Anopheles gambiae*

**DOI:** 10.1186/1475-2875-6-113

**Published:** 2007-08-21

**Authors:** Hortance Manda, Louis C Gouagna, Woodbridge A Foster, Robert R Jackson, John C Beier, John I Githure, Ahmed Hassanali

**Affiliations:** 1 International Centre of Insect Physiology and Ecology (ICIPE), PO Box 30772, Nairobi, Kenya; 2 Department of Biological Sciences, Kenyatta University, PO Box 43844, Nairobi, Kenya; 3 Département Société et Santé – UR016, Institut de Recherche pour le Développement (IRD), PO Box 182, Ouagadougou 01, Burkina Faso; 4 Department of Entomology, The Ohio State University, Columbus, Ohio, USA; 5 School of Biological Sciences, University of Canterbury, Christchurch, New Zealand; 6 Department of Epidemiology and Public Health, University of Miami, Miami, Florida, USA

## Abstract

**Background:**

A previous study showed for *Anopheles gambiae s.s*. a gradation of feeding preference on common plant species growing in a malaria holoendemic area in western Kenya. The present follow-up study determines whether there is a relationship between the mosquito's preferences and its survival and fecundity.

**Methods:**

Groups of mosquitoes were separately given *ad libitum *opportunity to feed on five of the more preferred plant species (*Hamelia patens*, *Parthenium hysterophorus*, *Ricinus communis*, *Senna didymobotrya*, and *Tecoma stans*) and one of the less preferred species (*Lantana camara*). The mosquitoes were monitored daily for survival. Sugar solution (glucose 6%) and water were used as controls. In addition, the fecundity of mosquitoes on each plant after (i) only one blood meal (number of eggs oviposited), and (ii) after three consecutive blood meals (proportion of females ovipositing, number of eggs oviposited and hatchability of eggs), was determined. The composition and concentration of sugar in the fed-on parts of each plant species were determined using gas chromatography. Using SAS statistical package, tests for significant difference of the fitness values between mosquitoes exposed to different plant species were conducted.

**Results and Conclusion:**

*Anopheles gambiae *that had fed on four of the five more preferred plant species (*T. stans*, *S. didymobotrya*, *R. communis *and *H. patens*, but not *P. hysterophorus*) lived longer and laid more eggs after one blood meal, when compared with *An. gambiae *that had fed on the least preferred plant species *L. camara*. When given three consecutive blood-meals, the percentage of females that oviposited, but not the number of eggs laid, was significantly higher for mosquitoes that had previously fed on the four more preferred plant species. Total sugar concentration in the preferred plant parts was significantly correlated with survival and with the proportion of females that laid eggs. This effect was associated mainly with three sugar types, namely glucose, fructose, and gulose. Except for *P. hysterophorus*, the results suggest that feeding by mosquitoes on preferred plant species under natural conditions results in higher fitness-related benefits, and that the sugar content in preferred plant parts is largely responsible for these effects.

## Background

For most mosquito species, floral nectar and extra-floral plant fluids are the female's primary source of dietary sugar and the male's only source of nutrients [1,2]. Yet the influence of plant feeding on mosquito fitness remains poorly understood, and this may be a serious gap in the knowledge of factors that affect the biotic potential, population dynamics and habitat suitability of *Anopheles gambiae s.s*., the primary vector of *Plasmodium falciparum *in sub-Saharan Africa [3-5]. Laboratory studies suggest that sugar meals may have an important influence on the flight performance, survival, and fecundity of adult mosquitoes [6,7]. However, interpreting findings from laboratory studies on *An. gambiae *has not been straightforward because, although females live longer when sugar is available [8-11], their daily fecundity is lower [9], apparently because of delayed onset or completion of oviposition [12,13].

Current knowledge of the effects of plant-mosquito interactions on fitness is fragmented, with respect to both experimental conditions and the component of fitness that is affected most. For instance, only two previous studies have focused on the survival of *An. gambiae *when feeding on plants [14,15]. However, the plants used were selected arbitrarily. One crucial question that has remained largely unaddressed, however, concerns the effect of sugar meals on mosquito survivorship and reproductive success in the presence versus the absence of preferred sugar sources. From an evolutionary standpoint, plants with high sugar content may confer correspondingly high survival reward and can be assumed to be more attractive to mosquitoes than plants with lower sugar content. In a recent study [16], feeding preferences of *An. gambiae *and the plant parts they preferred feeding on were ranked. This was done by giving the mosquitoes access to 13 of the dominant plant species growing in western Kenya. Testing was done in choice (i.e. with all the plant species present simultaneously) and in no-choice (i.e. with one plant species present at a time) situations. The present study proposes that *An. gambiae*'s preference ranking of plants matches the relative fitness-related benefits the mosquito derives from feeding on the different plant species. Survival and fecundity are specifically considered here, these being widely recognized as especially relevant to fitness. In addition, to assess plant-related influences on the fitness of *An. gambiae*, the quality and quantity of sugar from the plants tested were measured and the association of these variables with mosquito survival and fecundity were examined. The initial hypothesis of this study was that *An. gambiae *has evolved an ability to identify and feed preferentially on plant species that have especially high sugar content, with these, in turn, being plants on which *An. gambiae *has higher survival and fecundity.

## Materials and methods

### Study area

The study was carried out on the shores of Lake Victoria at the Thomas Odhiambo Campus (TOC) of the International Centre of Insect Physiology and Ecology (ICIPE) in Mbita Point, a village of about 8,000 people (primarily fishermen and traditional farmers) in Suba District, western Kenya. This is a locality of holoendemic malaria with about five infective bites per person per year [17,18]. Although *Anopheles arabiensis *and *Anopheles funestus *are also significant vectors of *P. falciparum *in Mbita Point, the primary vector is *An. gambiae s.s*. [17,18]. *Plasmodium falciparum *malaria is the leading cause of morbidity for residents in the area, accounting for 50–60% of all clinically diagnosed illness at the local health centre [19]. Mean minimum and maximum daily temperatures are 17°C and 34°C, respectively. Annual rainfall is 700–1200 mm, coming primarily during two rainy seasons, March to May and October to November. Permanent and semi-permanent larval habitats for *An. gambiae *are widespread during the dry seasons. Vegetation, which includes a wide variety of indigenous and introduced plants, remains verdant all year. Various shrubs and herbaceous plants are usually found around larval habitats, human habitations, and roadsides, representing potential sugar sources for local mosquitoes.

### Mosquitoes

Mosquitoes used in this study were obtained from a colony reared at ambient temperature and humidity, established in 2001 from blood-fed and gravid *An. gambiae s.s*. caught in Mbita Point. Adults were kept in standard 30 × 30 × 30 cm mesh-covered cages at the ICIPE-TOC insectary and offered a 6% glucose solution *ad libitum*. Females were also allowed to feed on a human volunteer arm for 15 min on three consecutive nights per gonotrophic cycle. Approval for feeding the mosquitoes on human subjects was obtained from the Kenya National Ethical Review Board (protocol number KEMRI/RES/7/3/1). Fully engorged females were then allowed to lay eggs in oviposition cups (4 cm diameter, 2 cm depth) placed inside the cages. Eggs were collected the following day and dispensed into plastic trays (25 cm long × 20 cm wide × 14 cm high) filled to a depth of 8 cm with filtered water collected from Lake Victoria. Upon hatching, larvae were reared in these pans at densities of 100–150 per tray and fed fish food (Tetramin^®^) three times per day. For experiments, pupae were transferred to standard cages and the emerging adults were denied access to blood or sugar prior to experiments, which started on the second day after emergence.

### Assessment of plant-feeding success and mosquito preference for different plant species and plant parts

A previous study [16] determined *An. gambiae*'s feeding responses to 13 perennial plant species: *Cassia hirsuta*, *Datura stramonium*, *Flaveria trinervia*, *Hamelia patens*, *Ipomea hildebrandtie*, *Lantana camara*, *Parthenium hysterophorus*, *Psiada punctulata*, *Ricinus communis*, *Senna bicapsularis*, *Senna didymobotrya*, *Tecoma stans *and *Tithonia diversifolia*. These plants were selected on the basis of their local availability in the vicinity of human dwellings and larval habitats of *An. gambiae *within a radius of 30 km around Mbita Point in western Kenya. Groups of 100 or 200 mosquitoes were released into cages and left overnight either with cuttings (branches with inflorescences) of one plant type at a time (no-choice assay) or with cuttings of all 13 plants simultaneously (choice assay), respectively. In the choice assay, direct observations of the numbers and percentages of mosquitoes perching or feeding on each plant species and the plant parts were recorded over four one-hour periods each night. For both types of assay, mosquitoes were recaptured and the percentage that had fed on plants was assessed by testing them individually for the presence of fructose. To confirm the plants and plant parts mosquitoes had fed on during the choice-assays, gas chromatography (GC) profiles of a sub-sample of mosquito homogenates were compared with GC profiles of extracts from relevant parts of each plant. It was found that *An. gambiae *is differentially responsive to this range of plants, regardless of whether the plants were presented singly or mixed together. It was also found that significantly more females than males fed on the plants. A mean of 72% of the mosquitoes were observed feeding and 56% of all mosquitoes recaptured were positive for fructose per trial in the choice assay. Means of 60%, 4% and 8% were observed feeding on flowers, leaves and stems respectively, in the choice assay. For most plant species (ten of 13), GC profiles also indicated that *An. gambiae *obtained sugars primarily from flowers. The exceptions were *P. hysterophorus *L., *L. camara *L. and *R. communis *L., as *An. gambiae *fed from leaves and stems more often than from flowers of these three species.

### Plants

**Figure 1:**
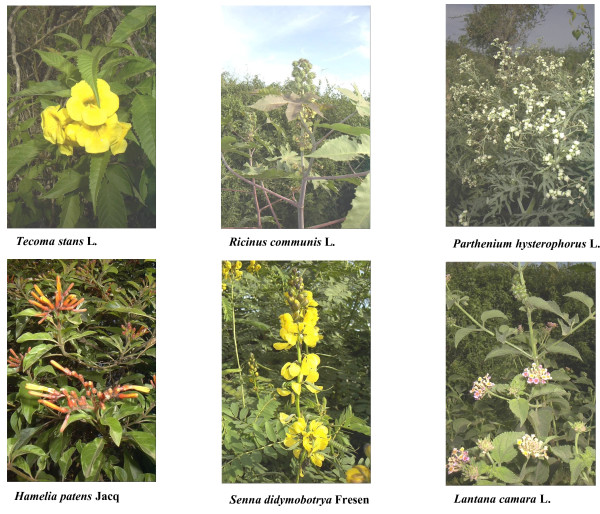
Plant species used in survival and fecundity study of *An. gambiae*.

Of the 13 plant species used in the study of *An. gambiae's *plant discrimination behaviour [16], six were selected for the present study (Table 1 and Figure 1). In that study, mosquitoes ranked four of these highest (*Parthenium hysterophorus *L., *Tecoma stans *L., *Ricinus communis *L., *Senna didymobotrya *F.) in both choice and no-choice testing. One (*Hamelia patens *J.), although ranked lower by mosquitoes in no-choice assay, was among the more preferred plant species in the choice assay. The other plant (*Lantana camara *L.) was a representative less-preferred species. When needed, cuttings (branches with inflorescences) were collected from the field and taken to the laboratory for testing with mosquitoes, care being taken not to damage the plants during transportation. All plants were cleared of potential predators (ants, spiders and other) before testing began.

**Table 1:** List and common names of plant species used in survival and fecundity study of *An. gambiae*

Family	Species	Common name
Rubiaceae	*Hamelia patens *Jacq	Firebush
Verbenaceae	*Lantana camara *L.	Wild sage
Asteraceae	*Parthenium hysterophorus *L.	Wild quinine
Euphorbiaceae	*Ricinus communis *L.	Castorbean
Leguminosae	*Senna didymobotrya *Fresen	African senna
Bignoniaceae	*Tecoma stans *L.	Yellow bells

### Survival assay

Batches of newly emerged (1-day old) mosquitoes, each consisting of 25 males and 25 females, were put in standard (30 × 30 × 30 cm) cages and placed in a screen-walled hazard-proof greenhouse (11.5 × 7.1 m). Randomly constituted groups of mosquitoes were exposed to each of the six plants species. In control groups, other batches of mosquitoes were allowed continuous access either to a 6% (wt:vol) glucose solution in filter-paper wicks (positive control) or water pads only (negative control). There was also a second negative control (a group deprived of sugar and of water). For each treatment group, cuttings with flowers from each of the six plant species were used, each being held in a 250 ml Erlenmeyer flask filled with fresh Lake Victoria water, the top being plugged with cotton wool and sealed with Parafilm^® ^(Pechiney Plastic Packaging, Menasha, WI, USA) to block the mosquito's access to the water. The experimental setup was exposed to fluctuating ambient temperature range of 18–31°C. Direct sunlight, hence extremes of temperature, was prevented by a layer of reed mats suspended beneath the glass roof of the experimental arena. Mosquitoes in each cage had access to one of the six plant species and to cotton pads wetted with distilled water. Plant material, glucose solution, and water pads were changed every two days. The survival of both female and male mosquitoes kept on different nutritional regimes was monitored until all the mosquitoes had died. Dead mosquitoes were removed and counted daily from each cage. All tests were replicated six times between April and December 2004.

### Fecundity assay

Two experiments were conducted for each plant species: a one-blood-meal (1-bm) experiment, replicated four times, and a three-blood-meal (3-bm) experiment, replicated seven times. For both, batches of 100 *An. gambiae *(50 females and 50 males) that emerged the previous night were released into each of eight standard cages. The rationale for putting males and females in each cage was to increase the chance that the females would be inseminated. In six cages, the mosquitoes were allowed access to each of the six plant species (one plant species per cage). In other two cages, mosquitoes were provided continuous access either to 6% (wt:vol) glucose solution in filter-paper wicks (positive control) or distilled water in cotton pads (negative control). In the 1-bm experiment, which began on the evening of the third day following exposure to plants, sugar or water, batches of female mosquitoes were allowed to feed on blood from a human arm (the same volunteer for each replicate) for 15 min. In the 3-bm experiment, female mosquitoes from each group were offered three successive blood meals (for 15 min each time) starting on the third day (i.e., blood meal offered on third, fourth and fifth evening). The 3-bm experiment was included because multiple blood meals have been shown previously to make vitellogenesis and egg maturation more likely to occur in *Anopheles *females [20]. Mosquitoes that failed to feed on blood were discarded after the first blood-feeding period. Immediately after blood feeding, and for the entire period of the assay, blood-fed mosquitoes were held in cages under their respective nutritional regimes. Based on previous experience, it was predicted that at least 90% of the mosquitoes surviving until the third evening after the start of the experiment would have fed at least once on the plant.

On the third evening following the one blood meal for the 1-bm experiment, or on the second evening following the third blood meal for the 3-bm experiment, gravid and hypergravid (delayed onset of oviposition) female mosquitoes were placed individually in smaller (15 × 15 × 15 cm) holding cages and given continual access to their respective food regimes. Plant cuttings were put in a 100 ml Erlenmeyer flask, filled with fresh lake water (access to the water in the flask prevented as indicated above). Food regimes were refreshed every 2 days.

In both the fecundity experiments, oviposition was allowed for 7 consecutive days by supplying individual mosquitoes with one plastic oviposition cup (4 cm diameter, 2 cm depth) containing 15 ml of distilled water and an inserted filter-paper cone over the top. During the 7-day period, although plants inside the cages were also checked for the presence of eggs, the mosquitoes always laid their eggs on the wet filter paper. Starting at 09.00 h each morning, each cup was changed and the number of eggs laid by each mosquito was counted under a dissection microscope. In the 3-bm experiment, dead mosquitoes found during the oviposition period were individually dissected, their ovaries were examined for the presence of retained eggs and the numbers of eggs were recorded. After the oviposition period, for each group the numbers of eggs retained were also determined by dissecting the remaining mosquitoes. The mean number of eggs produced by mosquitoes in each group was obtained by counting the number of eggs oviposited plus the number of eggs retained.

To determine egg viability, eggs laid by mosquitoes that have been exposed to the same nutritional regime were pooled and dispensed into plastic trays (25 cm long × 20 cm wide × 14 cm high) filled with filtered Lake Victoria water to a depth of 8 cm. First instar larvae were counted upon hatching.

### Extraction and quantification of sugars present in each plant

Using gas chromatography (GC), the sugar composition and the concentration of each sugar component in floral and extrafloral parts of each plant were determined. For each plant tested, parts (flowers and leaves) were extracted for analyses. Flowers included nectar and floral tissue. In the case of *R. communis*, stems (= petioles) were also included for analysis because mosquitoes were observed feeding from extrafloral nectaries on the stems of this plant species [16]. Using small cryo-preservation vials containing 200 μl of a 50% EDTA solution, approximately the same mass of flowers or leaves (or stems for *R. communis*) were incubated in the dark for 2 h, after which the plant material was removed and extracts remaining in the vials dried under a flow of pure nitrogen [21] and then analysed as described below.

### Sugar standards

Analytical grade sugars used as standards were obtained from Sigma-Aldrich Co. (Poole, Dorset, UK). Nine standards were prepared for derivatization and GC: α-D-glucose, D(+)raffinose, D(+)galactose, D(+)mannose, β-D-fructose, sucrose, D-gulose, D-altrose, and D-allose. For checking the analytical procedure, α-lactose was also included.

### Trimethylsilylation of standards and samples

Sugar analysis by GC requires initial silylation of the highly polar carboxyl and hydroxyl groups. Using previously described analytical procedures [21], sugar standards were trimethylsilylated in a clean 2-ml reacti-vial by dissolving 1 mg of each in 100 μl of dry pyridine. An equal volume of N-methyl-N-trimethylsilyltrifluoroacetanamide (MSTFA) was then added. The mixture was placed in an oven set at 60°C and left for 60 min, after which the sample was removed and stored at room temperature until analysis. Plant extracts were similarly processed and derivitized.

### Gas chromatographic analysis of sugars

These were performed on a Hewlett Packard (HP) (Walbronn Division B4, W-Germany) 5890 series II gas chromatograph, equipped with a split-less injector system, a 50 m × 0.2 mm (i.d.) crossed-linked methylsilicone (0.33 μm film thickness) capillary column and FID-coupled to an HP 3393 A Series II integrator. The carrier gas was nitrogen, with flow set at 0.8 ml/min. The initial temperature (100°C) was increased at 30°C/min up to 170°C and then at 2°C/min to 210°C, followed by 50°C/min to a final isothermal temperature of 280°C that was maintained for 30 min.

The derivatized sugar standards were diluted by a factor of 40 with dichloromethane (DCM 99.9+%, PRA grade). Three parts of the derivatized plant extract were diluted with one part of the same solvent, and 1 μL of each sample was injected into the gas chromatograph (using DCM to clean syringes between samples). Plant-derived sugars were identified by comparing retention times of sugar standards with those present in the plant extracts. The amount of each sugar in the plant samples was determined by accounting for the dilution factors and using the following formula:

Xi = (P_i_/P_s_) (X_s_)

where P_i _and P_s _are peak areas of sample and standard respectively, and X_s _the quantity of sugar standard injected.

### Statistical analyses

Survival analysis techniques (PROC LIFETEST), including a log-rank test, were used to compare survival curves and test whether the survival rate differed between different nutritional regimes and between the sexes. Effects of feeding treatment on survivorship were estimated using analysis of variance (ANOVA) on transformed (log_10_) data. For all measurements, means ± standard errors are given. Mean survival times were compared between groups using a Student-Newman-Keuls (SNK) test.

Data from the fecundity assay were either 1) log_10_-transformed for number of eggs produced, retained and laid per female in each nutritional regime or 2) arcsin-transformed for the percentages of mosquitoes that oviposited and for eggs that hatched. With ANOVA, the transformed data were analysed for effects of the nutritional regime, with means for these variables being compared separately using the SNK test.

The survival time and fecundity indices in mosquitoes exposed to different nutritional regimes were expressed relative to the positive control (glucose) as follows: value = (control-treatment)/control. Spearman correlation was used to detect potential linear relationships between sugar content of the plant parts preferred by the mosquitoes (flowers for *T. stans*, *S. didymobotrya *and *H. patens*, stems for *R. communis*, and leaves for *L. camara *and *P. hysterophorus*) [16] and mosquito median survival time and fecundity. Sugar concentration in flowers and leaves were compared using *t*-test. All mosquitoes that escaped or were inadvertently killed while handling were excluded from data analysis. For all analysis, the significance level was set at P ≤ 0.05. Analyses were carried out with Excel 2000^® ^and SAS version 8.2 for Windows^®^.

## Results

### How different plant species affected *An. gambiae*'s survival

Mean survival times of mosquitoes varied significantly among the nutritional regimes (F = 202.81, df = 8, P < 0.001). Mosquitoes that were given access to glucose solution (6%) survived significantly longer than mosquitoes from any other group. Survival time was shortest when mosquitoes were exposed to water alone (negative control) and to no water, glucose or plants (second negative control). Survival time on *P. hysterophorus *was comparable to survival times on the negative controls (Table 2). Survival time of mosquitoes on *L. camara*, the least preferred of the six plants investigated, was significantly higher than on the negative controls (P < 0.001 for each) or on *P. hysterophorus *(P < 0.001), and comparable to that of mosquitoes on *H. patens *(P = 0.2). However, survival time on *L. camara *was significantly lower than on the other plants or on the control (glucose) (P < 0.001 for each) (Table 2).

**Table 2:** Survival times in days of *An. gambiae *exposed to different nutritional regimes

Nutritional regime		N	Median	Mean	± SE	Range	Survival index	P
Glucose (6%)		300	16.0	17.1^a^	0.4	2.0 – 35.0	0.00*	NA
*T. stans*		294	12.0	13.4^b^	0.5	2.0 – 55.0	0.18^a^	0.06
*S. didymobotrya*		278	11.0	11.6^c^	0.3	2.0 – 34.0	0.30^a^	0.03
*R. communis*		293	10.0	11.4^c^	0.4	2.0 – 37.0	0.32^a^	0.01
*H. patens*		288	7.0	7.7^d^	0.3	2.0 – 32.0	0.50^b^	0.001
*L. camara*		286	6.0	7.2^d^	0.2	2.0 – 26.0	0.58^b^	<0.001
*P. hysterophorus*		290	4.0	4.7^e^	0.2	2.0 – 26.0	0.72^c^	<0.001
Water (-ve control)		297	3.0	3.8^e^	0.1	2.0 – 12.0	0.77^c^	<0.001
None (2^nd ^-ve control)		296	3.0	3.0^e^	0.0	2.0 – 6.0	0.80^c^	<0.001

N is total number of mosquitoes tested

SE is Standard error of mean

Survival index: (C-T)/C [(glucose control - treatment)/glucose control] expresses the effect of each treatment on the mean survival time of mosquitoes relative to the positive control (glucose)

Means and survival index with different letter superscripts in the same column are significantly different (SNK test at α = 0.05)

* Reference group

Overall, the ranking (from highest to lowest) of mean survival times of mosquitoes on the various plant species was as follows (Table 2): *T. stans*, *S. didymobotrya*, *R. communis*, *H. patens*, *L. camara *and *P. hysterophorus*.

Relative to the glucose solution, *T. stans*, *S. didymobotrya *and *R. communis *reduced survival of mosquitoes by 18%, 30% and 32%, respectively. *H. patens *(50%) was comparable to *L. camara *(58%), whereas *P. hysterophorus *and negative controls caused the highest reduction in survival (>70%).

**Figure 2:**
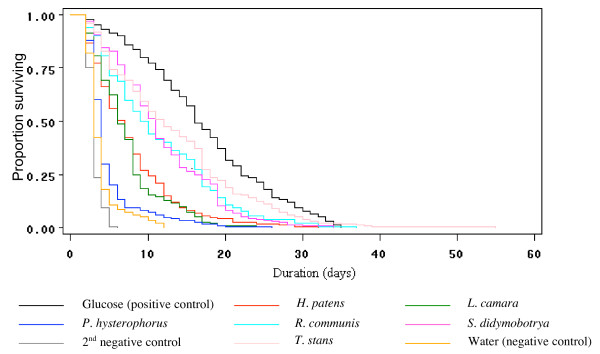
**Survival curves of *An. gambiae *(pooled males and females) exposed to different nutritional regimes**.

The proportion of mosquitoes surviving over time was influenced by feeding regime (χ^2 ^= 1633, df = 8, P < 0.001) (Figure 2). There were especially rapid declines in the proportions of surviving mosquitoes in the negative controls and in the *P. hysterophorus *groups, with numbers dropping dramatically during the first 5 days. Only 10% of the mosquitoes exposed to *P. hysterophorus *survived longer than 7 days. Survival rate also declined rapidly when mosquitoes were exposed to *L. camara*, with 80% being dead by day 10. Male and female mosquitoes that were exposed to 6% glucose solution or to *T. stans*, *R. communis *or *S. didymobotrya *had consistently higher survival rates than the other groups, and mortality rate was distributed more uniformly throughout the experimental period (Figure 2). There were no significant differences between the survival rates over time of males and females exposed to different plants (pooled replicates and pooled plants) (χ^2 ^= 0.28, df = 1, P = 0.63). Nor were there significant sex differences in mean survival times and survival curves found for individual plant species (P values, 0.34–0.94, Table 3).

**Table 3:** Sex differences in survival times and curves of *An. gambiae*, stratified by nutritional regime (log-rank test)

Nutritional regime	Males (N) Means ± SE (in days)	Females (N) Means ± SE (in days)	χ^2^*	P
Glucose (6%)	(148) 17.3 ± 0.6	(151) 17.2 ± 0.6	0.004	0.94
*T. stans*	(145) 14.2 ± 0.8	(154) 13.2 ± 0.6	0.89	0.34
*S. didymobotrya*	(131) 11.9 ± 0.5	(142) 11.4 ± 0.5	0.01	0.88
*R. communis*	(143) 10.9 ± 0.5	(142) 12.6 ± 0.6	3.07	0.07
*H. patens*	(136) 8.1 ± 0.4	(150) 7.4 ± 0.4	0.88	0.34
*L. camara*	(149) 7.6 ± 0.3	(145) 6.7 ± 0.3	2.93	0.08
*P. hysterophorus*	(138) 4.7 ± 0.3	(141) 4.7 ± 0.3	0.002	0.96
Water (-ve control)	(148) 3.8 ± 0.1	(149) 3.8 ± 0.1	0.01	0.90
None (2^nd ^-ve control)	(144) 2.9 ± 0.07	(143) 3.1 ± 0.07	2.58	0.10

* Test statistic for log-rank analysis: survival distribution between male and female mosquitoes in each respective nutritional regime

N is number of mosquitoes tested

SE is standard error of mean.

### How different plant species affected *An. gambiae*'s fecundity

When mosquitoes were offered only one blood meal, the number of eggs laid per mosquito varied significantly among groups exposed to the different plant species (F = 2.82, df = 7, P = 0.01). The numbers of eggs laid by mosquitoes that had been exposed to *T. stans, R. communis, S. didymobotrya *or *H. patens *were comparable to the number laid by mosquitoes that had fed on glucose. Mosquitoes from the *P. hysterophorus *and the *L. camara *groups laid significantly fewer eggs than mosquitoes from other groups, these being comparable to the number of eggs laid by mosquitoes from the negative control (Table 4).

**Table 4:** Fecundity of *An. gambiae *offered one blood meal and exposed to each nutritional regime

Nutritional Regime	N	Eggs laid Mean ± SE (female laying)
Glucose (6%)	44	35.7 ± 2.1^a^
*T. stans*	40	34.8 ± 2.0^a^
*S. didymobotrya*	46	33.1 ± 1.9^a^
*R. communis*	39	33.0 ± 2.3^a^
*H. patens*	48	39.1 ± 2.6^a^
*L. camara*	46	21.2 ± 2.5^b^
*P. hysterophorus*	38	15.1 ± 2.0^b^
Water	16	15.5 ± 3.2^b^

N is number of mosquitoes that oviposited

SE is standard error of mean

Any two means sharing a letter superscript in common are not significantly different (SNK test at α = 0.05)

For mosquitoes offered three consecutive blood meals, although there was no significant variation in the proportion of mosquitoes that produced eggs per group (F = 1.33, df = 7, P = 0.25), there was significant variation in the proportions of mosquitoes that oviposited (F = 3.38, df = 7, P = 0.006) and the proportions of mosquitoes that retained eggs (F = 2.90, df = 7, P = 0.014). Significantly fewer mosquitoes maintained on *P. hysterophorus, L. camara *or water (negative control) oviposited any eggs at all (Table 5). These three nutritional regimes reduced the percentage of mosquitoes ovipositing by more than 40% relative to the positive control (glucose solution). In the *T. stans*, *S. didymobotrya *or *R. communis *groups, proportions of mosquitoes ovipositing were comparable to the positive control, whereas that of mosquitoes from the *H. patens *group was intermediate (Table 5). However, there was no notable effect of diet on the numbers of eggs produced (Table 5). The numbers of eggs retained (F = 1.48, df = 6, P = 0.17) and laid (F = 1.29, df = 6, P = 0.29) per female were similar across plant species and controls (Table 5). Egg hatchability did not vary significantly among different nutritional groups (F = 1.68, df = 7, P = 0.14) (Table 5), suggesting no effect of sugar source on egg viability.

**Table 5:** Fecundity of *An. gambiae *offered three blood meals and exposed to each nutritional regime

Nutritional regime	N	Number of eggs produced Mean ± SE	Number of eggs retained Mean ± SE (per female retaining)	% Mosquitoes ovipositing Mean ± SE	Oviposition index	P	Number of eggs laid Mean ± SE (per female laying)	% Eggs hatched Mean ± SE
Glucose (6%)	117	76.5 ± 3.5^ab^	60.4 ± 5.2^a^	71.2 ± 6.7^a^	0*	NA	77.0 ± 4.6^a^	67.4 ± 1.2^a^
*T. stans*	115	63.4 ± 3.1^ab^	50.1 ± 4.0^a^	69.2 ± 6.7^a^	0.02^a^	0.85	67.4 ± 3.9^a^	69.0 ± 6.8^a^
*S. didymobotrya*	115	70.1 ± 3.2^ab^	63.1 ± 5.0^a^	64.5 ± 6.8^ab^	0.09^a^	0.78	68.0 ± 4.2^a^	61.9 ± 3.3^a^
*R. communis*	90	78.2 ± 3.7^a^	67.4 ± 5.5^a^	60.5 ± 7.3^ab^	0.15^a^	0.75	79.3 ± 4.7^a^	58.3 ± 7.7^a^
*H. patens*	95	66.0 ± 3.5^ab^	60.5 ± 4.5^a^	46.8 ± 8.0^bc^	0.34^b^	0.46	67.7 ± 5.8^a^	67.3 ± 9.1^a^
*L. camara*	82	70.6 ± 3.8^ab^	65.0 ± 5.8^a^	40.2 ± 7.6^c^	0.43^bc^	0.03	75.6 ± 5.1^a^	67.8 ± 4.1^a^
*P. hysterophorus*	80	60.4 ± 3.6^b^	53.7 ± 6.1^a^	39.1 ± 10.7^c^	0.45^bc^	0.02	57.8 ± 4.1^a^	34.4 ± 4.9^a^
Water	69	66.7 ± 3.9^ab^	67.2 ± 4.8^a^	27.5 ± 6.8^c^	0.60^c^	0.007	58.5 ± 6.1^a^	61.6 ± 17.4^a^

N is number of mosquitoes tested; SE is standard error of mean

Oviposition index: (C-T)/C [(glucose control - treatment)/glucose control] expresses the effect of each treatment on the percentage of mosquitoes ovipositing, relative to the positive control (glucose)

Any two means in the same column sharing a letter superscript in common are not significantly different (SNK test at α = 0.05)

* Reference group

### Sugar composition and concentration in plant extracts and correlation with mosquito survival and fecundity

Sugar composition and concentration varied among the plant species (Table 6). Of the sugars tested, α-D-glucose, β-D-fructose, sucrose, D(+)mannose, and D-gulose were dominant in all species. Across plant species, sugar tended to be more concentrated in flowers than in leaves (*t *= 2.48, *P *= 0.01 after pooling data across plant species) (Table 6). The highest sugar diversity (78% of sugars tested) and the highest total concentration of sugar (506 μg/mg of dry extract) were found in the flowers of *T. stans*. The lowest sugar diversity (22% of sugars tested) and the lowest total concentration (2 μg/mg of dry extract) were found in the leaves of *P. hysterophorus*, the flowers of this plant having no identifiable sugars at all.

**Table 6:** Sugars present in each plant species and quantity in 1 mg dried extract of plant parts

Plants	Plant part	α-D-Glucose (μg)	β-D-Fructose (μg)	Sucrose (μg)	D-Mannose (μg)	D-Gulose (μg)	D-Galactose (μg)	D-Raffinose (μg)	D-Altrose (μg)	D-Allose (μg)	Total (μg)
*H. patens*	Flower	5.1	0	12.8	3.0	0	0	0	0	0	20.9
	Leaf	0	0	0	0	0	0	0	0	0	0
*L. camara*	Flower	43.8	30.6	2.4	8.6	134.6	0	0	0	0	220
	Leaf	4.6	0	0	0	0	0	0	0	0	4.6
*R. communis*	Flower	23.3	0	0	2.2	0	0	0	0	0	25.5
	Stem	7.3	15.2	0	0	0	0	0	0	0	22.5
	Leaf	0	0	0	13.4	12.4	0	0	0	0	25.8
*S. didymobotrya*	Flower	78.8	5.4	2.5	14.8	110.8	7.3	0	0	0	219.6
	Leaf	2.4	10.5	1.1	0	38.9	0	0	0	0.9	53.8
*P. hysterophorus*	Flower	0	0	0	0	0	0	0	0	0	0
	Leaf	0	0	1.2	0	0	0.8	0	0	0	2
*T. sta*ns	Flower	58.5	161.7	0.3	3.9	263.3	0	3.95	14.5	0	506.2
	Leaf	2.0	0	0	0	0	0	0	0	0	2.0
r		0.94	0.88	0.02	0.75	0.84	-0.06	0.65	0.65	NA	1
Probability		0.004*	0.02*	0.95	0.08	0.03*	0.89	0.15	0.15	NA	<0.001*

r is Spearman coefficient of correlation between median survival time of mosquitoes and sugars in preferred parts of plants

* P ≤ 0.05 (significant association of sugar with median survival time).

NA is non applicable

There was a significant positive correlation between the total amount of sugar present in the preferred part of each plant species [16] and median survival time of mosquitoes exposed to the plants (r = 1.00, P < 0.001), and between each of the three types of sugars (glucose, fructose, and gulose) and mosquito survival (Table 6).

For mosquito fecundity, there was a positive, but not significant, correlation between the total sugar concentration in preferred feeding parts of plants and numbers of eggs laid when mosquitoes were offered one blood meal (r = 0.65, P = 0.15). The same result was obtained when mosquitoes were offered three blood meals (r = 0.14, P = 0.78). Nevertheless, the coefficient of correlation was clearly higher with one blood meal. There was a significant correlation between the proportion of mosquitoes ovipositing and sugar concentrations in plants (r = 1.00, P < 0.001).

## Discussion

In a previous study, *An. gambiae *in western Kenya was demonstrated to have a preference ranking for feeding on different plants and plant parts [16]. The objective of the present study was to take a subset of these plants and determine whether data on the mosquito's survival and fecundity form a ranking that corresponds to the previously determined feeding-preference ranking.

With the exception of mosquitoes exposed to *P. hysterophorus*, the ranking of the survival and of the fecundity of mosquitoes following exposure to plants was remarkably similar to the previously established feeding preferences. These values were also consistent with total sugar concentration and with the concentrations of three individual sugars (glucose, fructose and gulose) in the sites on these plants at which the mosquitoes especially often fed (flowers for *T. stans*, *S. didymobotrya *and *H. patens*, stems for *R. communis*, and leaves for *L. camara *and *P. hysterophorus*) [16]. These findings suggest that the sugar content in the different plant parts is approximately proportional to the amount of sugar available to the mosquitoes. Because a substantial proportion of a mosquito's energy reserves come from the sugar it consumes [22], variation among plant species in sugar content may potentially affect mosquito fitness. This study has shown that the choice of sugar source may be determined to a large extent by plant attributes including the particular types of sugar present and their concentration.

Besides fructose, glucose, mannose, sucrose, raffinose and galactose, gulose and allose were also detected, two sugars that have not been reported in previous analyses of nectar [23-25]. In the previous study [16], these sugars were also detected in the guts of *An. gambiae*, suggesting that these monosaccharides may occur in floral and other plant tissue. Detailed observations on the feeding behaviour of the mosquito and analyses of the plant parts fed upon may provide further insight into the general properties underlying the preference process and the related fitness gain.

*Lantana camara *is a special case. Despite the flowers of this plant being rich in sugar and the leaves being poor in sugar, *An. gambiae *fed mainly on *L. camara*'s leaves [16]. An explanation for this may be related to the long corollas of *L. camara*'s flowers, perhaps making the nectar in the flowers of this plant species inaccessible to mosquitoes, forcing *An. gambiae *to feed on the less nutritious leaves. This in turn might help to explain *L. camara'*s low position in *An. gambiae*'s feeding-preference ranking observed in the previous study [16], as well as its poor performance on this plant species, as reported also by other previous studies [14,15].

In view of *An. gambiae's *high preference-ranking for *P. hysterophorus *[16], this plant species is another special case, as the findings of this study go against what was expected. In the earlier study, the mosquitoes were seen frequently applying the tip of the proboscis to this plant's parts [16], but the poor survival and fecundity of mosquitoes on this plant species calls towards reconsidering interpreting this behaviour as instances of the mosquito feeding on plant-derived sugar. The flowers of *P. hysterophorus *had no detectable monosaccharides and only a low level of the disaccharide sucrose was found in the leaves of this plant. The mosquito's performance (survival on the plant and fecundity following one blood meal) on *P. hysterophorus *was comparable to its performance on the negative control. On the whole, the findings from the previous study [16] combined with those from the present study suggest a hypothesis currently being investigated: that with *P. hysterophorus*, there are plant constituents other than sugars that may be beneficial to the mosquito in hitherto unrecognized ways.

In the experiment where mosquitoes were offered three blood meals, there was no evidence of plant feeding affecting the number of eggs produced or oviposited by individual females. However, except for the *P. hysterophorus *group, the percentages of females that oviposited were significantly higher for the mosquitoes that had fed on the more preferred plants. This indicates that, although multiple blood meals can compensate for sugar [9,26,27], initial access to sugar has an advantageous effect on the *An. gambiae*'s fecundity even when repeated access to blood meals becomes possible.

On the whole, these findings suggest that the complexity and importance of mosquito-plant relationships are not as widely appreciated as they should be. For the mosquito, there appear to be distinct benefits when feeding on the floral and extrafloral nectar of different plant species. Variation in sugar content appears to be the primary factor accounting for fitness-related effects of different plant species on the mosquito's survival and fecundity. However, there is also evidence that, with some plants, *An. gambiae *may also frequently ingest plant-origin material from leaves [16] and perhaps from plant parts other than floral and extrafloral nectaries. Ingestion of plant-origin material other than nectar may be a general phenomenon in mosquitoes, as examinations of the midguts of *Culex pipiens molestus*, *Anopheles sergentii*, *Anopheles claviger *and *Aedes caspius *(Diptera: Culicidae) have revealed the presence of plant tissues [28,29]. This study underlines that access to sugar-rich plants can be indispensable to mosquito survival and fecundity, with the mosquitoes tending to get sugar from floral and extrafloral nectaries. However, there is now a reluctance to call all instances of a mosquito ingesting plant-origin material 'feeding' because the word 'feeding' implies something to do with nutrition and it is not clearly the case that obtaining nutrients is always relevant when plant-origin material is ingested. The results of the present study presented two examples of plant species from which *An. gambiae *ingested sugar primarily from the leaves [16], and the data from these two plant species resulted in two contrasting situations. In the case of *L. camara*, mosquitoes fed especially from the plant's leaves [16] where sugar content is low. Their poor performance on this plant is consistent with its low preference ranking in the earlier study [16]. *Anopheles gambiae's *strong preference for *P. hysterophorus *[16], despite poor performance on this plant, appears to be more paradoxical, and current studies underway may clarify the adaptive significance of *An. gambiae*'s preference for this plant species.

## Authors' contributions

HM participated in the design of the study, conducted all the experimental work, analysed and summarized the data and drafted the manuscript. LCG co-designed and coordinated the study, supervised the experimental work, analysis and summary of the data, and contributed to the manuscript. JIG, AH, JCB co-designed and co-ordinated the work, and contributed to the manuscript. WAF co-designed and contributed to the manuscript. RRJ contributed to the manuscript. All authors actively contributed to the interpretation of the findings, read and approved the final manuscript.
